# DNA damage complexity as a predictor of cell survival: a microscopic Monte Carlo-based modeling framework for photon, proton and carbon ion irradiation

**DOI:** 10.1088/1361-6560/ae7baa

**Published:** 2026-06-25

**Authors:** Miao Qi, Youfang Lai, Fada Guan, Meiying Xing, Lawrence Bronk, Radhe Mohan, Heng Li, Yujie Chi, Xun Jia

**Affiliations:** 1 Department of Radiation Oncology and Molecular Radiation Sciences, Johns Hopkins University, Baltimore, MD 21287, United States of America; 2 Department of Radiation Physics, University of Texas MD Anderson Cancer Center, Houston, TX 77030, United States of America; 3 Department of Radiation Oncology, University of Texas Southwestern Medical Center, Dallas, TX 75287, United States of America; 4 Department of Physics, University of Texas at Arlington, Arlington, TX 76019, United States of America

**Keywords:** Monte Carlo, DNA, radiation, cell survival

## Abstract

*Objective.* Biological effect of charged particles characterized by cell surviving fraction (SF) depends on physical factors such as particle type, dose, beam quality descriptors, e.g. linear energy transfer or lineal energy. Driven by the crucial role of DNA double-strand breaks (DSBs) as indicators of cellular responses to ionizing radiation, this study aims to develop a cell-specific SF model based on the initial DSBs of varying complexities applicable to photon, proton, and carbon-ion irradiations across the investigated beam qualities. *Approach.* Microscopic Monte Carlo simulations were used to calculate DSBs for experiments measuring SF of H460 and H1437 lung cancer cells irradiated by proton (dose-mean lineal energy ${\bar y_{\mathrm{d}}}$ 2.0–20.0 keV *µ*m^−1^), carbon ions (${\bar y_{\mathrm{d}}}$ 18.6–87.9 keV *µ*m^−1^) and reference ^137^Cs photon beam. We modeled SF as a third-order polynomial function of DSBs of various complexities with two, three, and more strand breaks. The function was determined by a data fitting process. *Main Results.* For each cell line, a single model was able to accurately describe SF in experiments under all 19 irradiation conditions with different particle types and ${\bar y_{\mathrm{d}}}$ values. Root mean square errors were 0.218 for H460 cells and 0.170 for H1437 cells. When applying the model to a proton spread-out-Bragg-peak case, calculated relative biological effectiveness at SF = 0.1 were 1.01–1.42 for the H460 cells and 1.03–1.65 for the H1437 cells depending on the depth. *Significance.* We successfully developed an SF model using DSBs of various complexities as input variables. The model provides a unified description of photon, proton, and carbon-ion irradiations across investigated beam qualities.

## 1. Introduction

In proton and carbon-ion therapy, cellular response to radiation, i.e. surviving fraction (SF), is affected by radiation quality characterized by linear energy transfer (LET) or microdosimetric quantity lineal energy. The concept of relative biological effectiveness (RBE) was introduced to account for the difference in physical dose to achieve the same SF between a particle beam and a reference photon beam, so that treatment protocols in the particle beam could be based on experience from the conventional photon radiotherapy (Paganetti *et al* 2002, Rørvik *et al* 2017). It is of critical importance to understand the variation of SF to better account for RBE in particle therapy, particularly at the distal end of the Bragg peak, where the beam quality changes rapidly and the increased RBE lead to concerns of normal tissue toxicity (Peeler *et al* 2016, Underwood *et al* 2018).

Over the years, extensive research has been conducted on modeling radiobiological effects. A straightforward approach involves linear regression between RBE and LET (Sørensen *et al* 2011, Paganetti 2014). In the linear-quadratic (LQ) formalism, model parameters $\alpha $ and $\beta $ are assumed to depend on macroscopic physics properties, such as dose-averaged LET, ${\mathrm{LET}}_d$ (Carabe *et al* 2013, Wedenberg *et al* 2013, McNamara *et al* 2015, Paganetti 2018), or microscopic quantities such as dose-mean lineal energy ${\bar y_d}$ (Hawkins 1998, Newpower *et al* 2019, Hartzell *et al* 2021). The LQ model then allows the calculation of SF and subsequently the RBE (Rørvik *et al* 2017). While these models effectively describe cellular SF behaviors, the lack of clear physical or biological justifications on the fitted functions, coupled with concerns about the validity of the LQ model in both low- and high-dose ranges, raises questions about the reliability of this approach.

Considering that DNA is the primary lethal target of radiation, the quantity and complexity of DNA damage are expected to serve as indicators for SF (Nikjoo *et al* 2008). Feasibility of this method is further supported by advances in microscopic Monte Carlo simulations (MMCSs), which can compute DNA damage based on fundamental principles (Nikjoo *et al* 1997, Friedland *et al* 2008, Incerti *et al* 2010a). These simulations inherently account for factors influencing DNA damage including not only dose but also particle track structure that affects damage complexity. Additionally, factors that modulate the chemical stage of radiation, such as the presence of oxygen, can also be incorporated (Incerti *et al* 2010b, Kyriakou *et al* 2016, Nikjoo *et al* 2016, Forster *et al* 2018, Matsuya *et al* 2020, Lai *et al* 2022).

Along this direction, Matsuya *et al* utilized the quotient of single-strand break (SSB) and double-strand break (DSB) yields for proton versus photon irradiation as a representation of proton RBE (Matsuya *et al* 2022). Henthorn *et al* developed a model for DNA repair based on simulated DNA damage distributions, establishing correlations between RBE and residual or misrepaired DSBs, along with LET metrics of various averaging forms (Henthorn *et al* 2018). However, due to the absence of radiation chemical stage modeling, these simulations did not account for the impact of indirect DNA damage. Bertolet *et al* simulated multiple monoenergetic irradiation scenarios, deriving a Gamma distribution-based relationship between the number and complexity of DNA damage and microdosimetric lineal energy (Bertolet *et al* 2023). SF and RBE could be estimated by incorporating a simple DNA repair model. Meanwhile, several studies have extended DNA damage-based SF modeling approaches through the integration of mechanistic and multiscale frameworks. For example, the Geant4-DNA–based ‘dsbandrepair’ tool integrates DNA damage simulations with established radiobiological models such as two-lesion kinetics (TLK) and LEM-IV models to estimate survival-related endpoints (Tuan Anh *et al* 2024). Similarly, Sakata *et al* incorporated the TLK model into a Geant4-DNA framework to calculate both DNA rejoining kinetics and cell survival for proton-irradiated V79 cells (Sakata *et al* 2023). Bordieri *et al* further developed the MT-GSM2 framework, which combines nanodosimetric DNA damage, microdosimetric energy deposition, and mechanistic lesion-evolution dynamics to calculate survival and RBE (Bordieri *et al* 2025). While these frameworks are highly valuable and provide strong mechanistic interpretability, they often involve substantial computational cost, require detailed assumptions on repair pathways, and extensive endpoint-specific data for robust calibration, which may limit their generalizability.

In this work, we aim to model SF behaviors in photon, proton, and carbon-ion irradiations directly from the numbers and complexities of initial DNA DSBs. The term ‘initial DSBs’ refers to those generated immediately after irradiation and not affected by the subsequent DNA repair process. We hypothesized that the cell SF behavior for a given cell type is a function of the initial DNA DSBs. This function is applicable across the investigated irradiation conditions, including different particle type, dose, LET (or lineal energy), because the function is expected to be intrinsic to the cell. This approach allowed us to employ the computation of DNA damage via MMCS to model cell SF, while avoiding explicit modeling of the complex biological responses in the DNA repair process. To address potential computational challenges, we used a GPU-based MMCS package, gMicroMC, to improve computational efficiency (Tsai *et al* 2020, Lai *et al* 2020, 2021b).

## 2. Methods

### 2.1. Overview of the proposed model

DNA damage repair is a complex process. It can be expected that, once the initial DNA damage patterns (number and complexity) emerge at the end of the radiochemical stage, the repair process only depends on the existing damages but not the form of radiation causing these damages. Hence, it is our goal to use the initial DNA damages as independent variables to calculate cell SF. We specifically focused on DSBs of various complexities due to their primary role in triggering cell responses. In this study, a strand break is a damage in a sugar phosphate group caused either by the accumulation of physical energy in the physical stage or reactions with radicals in the chemical stage. We denoted ${\mathrm{DSB}}_n$ as a cluster including a sequence of *n* strand breaks with no more than 10 base pairs between any adjacent strand breaks and these strand breaks exist on both strands, and $DS{B_{n + }}$ a cluster of at least n strand breaks. This classification is conceptually consistent with the treatment of simple and complex DSBs in the TLK model (Stewart 2001). In the present work, complex DSBs were further stratified into $DS{B_3}$, representing clusters containing three strand breaks, and $DS{B_{4 + }}$, representing clusters containing four or more strand breaks, to better resolve variations in damage severity relevant to cell survival outcomes.

**Figure 1.**
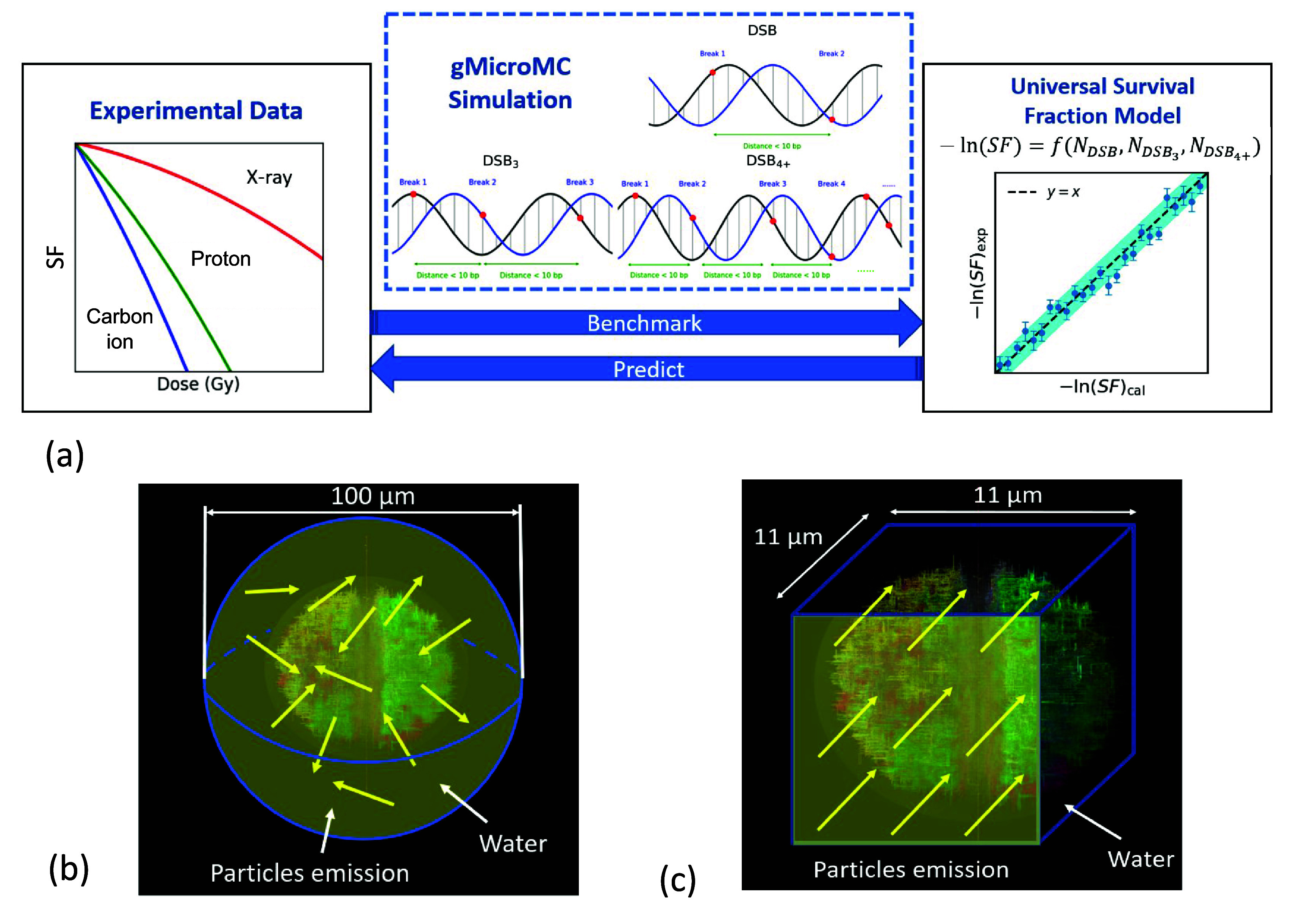
(a) Workflow of this study. MMCS was employed in computing DSBs under given irradiation conditions. With experimental SF data under different doses and particle irradiation, an SF model was built for the investigated irradiation conditions. (b) Illustration of simulation geometry for the photon irradiation case. (c) Simulation geometry for the proton and carbon-ion irradiation case.

The overall idea of the proposed SF model is illustrated in figure 1(a). Specifically, we wrote

\begin{align*} -{\text{ln SF}} = f\left( {{N_{{\mathrm{DSB}}}},{N_{{\mathrm{DS}}{{\mathrm{B}}_{\mathrm{3}}}}},{N_{{\mathrm{DS}}{{\mathrm{B}}_{{\text{4 + }}}}}}} \right){\text{ }}\end{align*}

where $f\left( . \right)$ is a cell-specific function mapping the numbers of different types of initial DSBs, ${N_{\mathrm{DSB}}},{N_{\mathrm{DSB}_3}},$ and ${N_{\mathrm{DSB}_{4 + }}}$, to SF. Since the specific form of $f\left( . \right)$ is unknown, we represented it with a third-order polynomial function including three linear terms, six quadratic terms, and one third-order term $N_{\mathrm{DSB}}^3$, which was empirically found to be sufficient. The specific function form is

\begin{align*}f\left( {{N_{{\mathrm{DSB}}}},{N_{{\mathrm{DS}}{{\mathrm{B}}_{\mathrm{3}}}}},{N_{{\mathrm{DS}}{{\mathrm{B}}_{{\text{4 + }}}}}}} \right) &amp; = {a_1}{N_{{\mathrm{DSB}}}} + {a_2}{N_{{\mathrm{DS}}{{\mathrm{B}}_{\mathrm{3}}}}} + {a_3}{N_{{\mathrm{DS}}{{\mathrm{B}}_{{\text{4 + }}}}}} + {a_4}N_{{\mathrm{DSB}}}^2 + {a_5}N_{{\mathrm{DS}}{{\mathrm{B}}_{\mathrm{3}}}}^2 + {a_6}N_{{\mathrm{DS}}{{\mathrm{B}}_{{\text{4 + }}}}}^2 \nonumber\\ &amp; \quad + {a_7}{N_{{\mathrm{DSB}}}}{N_{{\mathrm{DS}}{{\mathrm{B}}_{\mathrm{3}}}}} + {a_8}{N_{{\mathrm{DSB}}}}{N_{{\mathrm{DS}}{{\mathrm{B}}_{{\text{4 + }}}}}} + {a_9}{N_{{\mathrm{DS}}{{\mathrm{B}}_{\mathrm{3}}}}}{N_{{\mathrm{DS}}{{\mathrm{B}}_{{\text{4 + }}}}}} + {a_{10}}N_{{\mathrm{DSB}}}^3\end{align*}

where coefficient ${a_1}$–${a_{10}}$ are to be determined via model fitting. This form was adopted to take advantage of the flexibility of the polynomial function family for trend approximation, while simultaneously ensuring model simplicity and avoiding overfitting. Nonlinear terms were incorporated to consider interactions between different DSB complexities. For instance, the term ${N_{\mathrm{DSB}}}{N_{\mathrm{DSB}_3}}$ may represent the resultant chromosome change due to one DSB and another DNA DSB cluster.

### 2.2. MMCSs and DNA damage calculations

#### 2.2.1. DNA damage simulation

For efficiency consideration, we used GPU-based MMCS, gMicroMC, to compute DSBs in a multi-scale DNA geometry model (Tsai *et al* 2020, Lai *et al* 2020, 2021b, 2022). Radiation-induced physics interactions and water radiolysis with the presence of DNA were simulated, and DSBs of various complexities were computed (Lai *et al* 2021a, 2021b, 2022). These processes can be separated into three sequential stages: physical, physicochemical, and chemical.

In the physical stage, primary radiation particles and their secondary electrons were transported step by step in water. Each interaction was logged with its position and whether it resulted in ionization or excitation. DNA strand damage was determined based on energy deposition: if a physical event occurred within a reaction distance of 0.1 nm, which represents the thickness of the first hydration layer, from a sugar-phosphate group, the deposited energy was accumulated to that group. A strand break was recorded if the cumulative energy deposition reached or exceeded 17.5 eV.

In the subsequent physicochemical stage, excited or ionized water molecules decayed via predefined reaction pathways, generating initial positions of free radicals such as hydroxyl radicals and hydrated electrons.

In the chemical stage, we applied the concurrent transport method to simulate real-time interactions between diffusing radicals and DNA, allowing for ongoing reactions during radical diffusion. Two types of chemical reactions were modeled: (1) damage to DNA bases or sugar-phosphate groups by hydroxyl radicals and hydrated electrons, and (2) scavenging of radicals by histone proteins. A hydroxyl radical could induce a strand break if it approached within a reaction radius ${R_{c{\text{ }}}}$ of a sugar-phosphate group, with a reaction probability being $0.1$. The reaction radius ${R_c}$ is given by: ${R_c} = \frac{k}{{4\pi {N_A}D}}$, where *k* is the reaction rate, ${N_A}$ is the Avogadro constant, and $D$ is the diffusion rate for the radical. The chemical stage was simulated over 2.5ns, corresponding to the typical lifetime of hydroxyl radicals in cells.

Throughout the simulation, spatial coordinates of DNA strand breaks—whether caused by direct ionization (physical stage) or radical interactions (chemical stage)—were recorded. A sequential scanning procedure was then applied to identify clusters consisting of strand breaks on both DNA strands. Starting from a given strand break, subsequent strand breaks were examined to determine whether they satisfied the clustering criterion (i.e. within 10 base pairs). If strand breaks on both DNA strands were identified within this distance, a DSB candidate was formed. Additional strand breaks within the same spatial window were further examined to determine whether the cluster satisfied the criteria for higher-complexity categories. Three categories (figure 1(a)) of DSB clusters were scored:

• ${\mathrm{DSB}}$: an isolated double-strand break with only two strand breaks.
• ${\mathrm{DSB}}_3$: a cluster of three strand breaks within 10 bp.
• ${\mathrm{DSB}}_{4 + }$: a cluster of four or more strand breaks within 10 bp.

The numbers of ${\mathrm{DSB}}$, ${\mathrm{DSB}}_3$, and ${\mathrm{DSB}}_{4 + }$ were recorded as ${N_{{\mathrm{DSB}}}},{\text{ }}{N_{{\mathrm{DSB}}}}_3$ and ${N_{{\mathrm{DSB}}}}_{4 + }$ respectively. Once a cluster was identified, all strand breaks within that cluster were assigned to the corresponding DSB category and excluded from further consideration. This ensured that each strand break was counted only once, and that the resulting ${\mathrm{DSB}}$, ${\mathrm{DS}}{{\mathrm{B}}_3}$, and ${\mathrm{DS}}{{\mathrm{B}}_{4 + }}$ categories were mutually exclusive. Repeated simulations were performed to achieve a relative uncertainty level less than 5% for ${N_{\mathrm{DSB}}},{\text{ }}{N_{\mathrm{DSB}_3}}$ and ${N_{\mathrm{DSB}_{4 + }}}$. Note that clusters containing four or more strand breaks were grouped into the ${\mathrm{DSB}}_{4 + }$ category because of their low statistical yield, which would otherwise introduce substantial uncertainty into the simulation results.

#### 2.2.2. Accumulating DNA damage with increasing dose

We sampled source particles one at a time. For each sampled particle, the full sequence of physical, physicochemical, and chemical processes described in section 2.2.1 was simulated. After each particle simulation, the spatial coordinates of all strand breaks were accumulated within the cell geometry. This cumulative record represents the combined contributions of direct and indirect mechanisms to the total DNA damage at a given absorbed dose. From these accumulated strand breaks, the yields of different DSB cluster categories (${\mathrm{DSB}}$, ${\mathrm{DSB}_3}$, and $DS{B_{4 + }}$ as defined in section 2.2.1) were updated. Thus, the resulting quantities ${N_{\mathrm{DSB}}}$, ${N_{DS{B_3}}}$, and ${N_{DS{B_{4 + }}}}$ reflected the dose-dependent accumulation of DNA damage and were subsequently used as independent variables in model fitting to estimate the calculated SF ($S{F_{\mathrm{cal}}}$).

#### 2.2.3. Considerations for photon, proton and carbon-ion irradiations

For the reference photon irradiation case with ^137^Cs, we started MMCS with secondary electrons as source particles. The simulation world was a sphere with a radius of ${r_{cell}} + R$ as shown in figure 1(b). Inner sphere with radius ${r_{cell}} = $ 5.5 *μ*m hosted the DNA geometry model, while a shell region around the DNA geometry model with a thickness $R = {\text{ }}$ 44.5 *μ*m ensured electron equilibrium at the DNA region. The electron energies were sampled from an energy spectrum obtained by performing Monte Carlo (MC) simulations of the ^137^Cs irradiation case in a water phantom. The electron positions were randomly sampled in the simulation world, and the initial directions were isotropically distributed. For the charged particle cases, the simulation world was a cube with a dimension of 11.0 *μ*m, with the DNA model in the middle. Particles were randomly sampled on one face of the cube as shown in figure 1(c). The initial energy of the particles was sampled from an energy spectrum depending on the specific simulation scenarios (Guan *et al* 2015). In the proton cases, non-proton secondary fragments were excluded, because their contribution to dose is negligible (Guan *et al* 2019), and their inclusion did not lead to a statistically significant difference in the resulting DNA damage yields (the additional simulations result are provided in section S1.2). In contrast, for carbon ions, the dominant secondary fragments were included to account for their substantial effects on the local energy deposition and resulting DNA damages (Krämer *et al* 2003). Further details on the simulation setups are provided in Section S1.

### 2.3. Model fitting for pristine beams

While $LE{T_d}$ is widely used, many studies have shown that it is insufficient to describe radiation quality (Guan *et al* 2015, 2024, Grün *et al* 2019), and that microdosimetric quantities, such as dose-mean lineal energy ${\bar y_d}$, better reflect nanometer-scale track structure and characterize the mixed radiation fields in heavy ion irradiation (Braby *et al* 2023, Suárez-garcía *et al* 2023). In this study, we therefore used ${\bar y_d}$ to characterize radiation quality. ${\bar y_d}$ was calculated using event-by-event energy deposition scoring within a spherical water volume representing a cell nucleus. For each MC history, the total energy deposited by primary and secondary particles was recorded and converted to lineal energy using the mean chord length formalism. The resulting lineal energy spectra were used to compute ${\bar y_d}$ as the first moment of the dose-weighted distribution. Further details of the microdosimetric calculation, including scoring geometry and spectrum construction, are provided in section S2.

We considered the high-throughput cell SF measurements with H460 cells and H1437 cells (Guan *et al* 2015, Bronk *et al* 2020) under a series of 19 experimental conditions, including ^137^Cs photon irradiation, 12 proton irradiations with ${\bar y_d}$ in 2.0–20.0 keV *μ*m^−1^ along the path of a 79.7 MeV pristine proton beam and 6 carbon-ion irradiations with ${\bar y_d}$ in 18.6–87.9 keV *μ*m^−1^ along the path of a 153.66 MeV u^−1^ C-12 ion beam.

For each data point with measured SF, $SF_{exp}^j$, where $j$ is the index of data, we computed corresponding numbers of DSBs ${N^j} = \left( {N_{{\mathrm{DSB}}}^j,N_{{\mathrm{DS}}{{\mathrm{B}}_{\mathrm{3}}}}^j,N_{{\mathrm{DS}}{{\mathrm{B}}_{{\text{4 + }}}}}^j} \right)$ based on the specific experiment condition. With the data, we determined the function $f\left( . \right)$ by minimizing a loss function $L\left( \theta \right)$:

\begin{align*}L\left( \theta \right) = \sum\limits_j {{W_j}} {\left[ {\ln {\mathrm{SF}\,}_{{\mathrm{exp}}}^j - f\left( {{N\,^j}{\mathrm{|}}\theta } \right)} \right]^2}H\left[ {\left| {{\mathrm{SF}\,}_{{\mathrm{exp}}}^j - \exp \left[ { - f\left( {{N\,^j}{\mathrm{|}}\theta } \right)} \right]} \right| - \Delta } \right] + \sum\limits_{i,j} {{\lambda _i}} {\left[ {{{\left. {\frac{{\partial f\left(N|\theta \right)}}{{\partial {N_i}}}} \right|}_{{N\,^j}}}} \right]^2}\end{align*}

where $\theta $ represents polynomial coefficients in $f\left( . \right)$ and $i = 1, \ldots ,3$ is the index for DSB complexity types. The first term ensures data fidelity between the experimental and the fitting results, and the second term regularizes the model smoothness with respect to the DNA damage numbers. ${W_j}{\text{ }}$ and ${\lambda _i}$ were empirically chosen to balance the magnitude of different terms. $H\left[ . \right]$ is the step function to avoid punishing discrepancy less than a threshold ${{\Delta }}$, allowing certain deviations considering the uncertainty associated with experiments.

To avoid overfitting, we conducted a cross-validation study. In each fold of the cross-validation, experimental data of 16 series for different ${\bar y_d}$ were chosen among the photon, proton and carbon-ion cases, and the remaining 3 series were used for validation, so there are 969 folds. The fitting and validation root mean square errors (RMSEs) among these folds were analyzed to assess the model’s generalizability with RMSE defined as ${\mathrm{RMSE}} = \sqrt {\frac{1}{M}\sum\limits_j | \ln {\mathrm{SF}}_{{\mathrm{exp}}}^j - f({N^j}|\theta ){|^2}} $, where $M$ is the number of data points.

Following model fitting and validation, additional analyses were performed using the resulting cell-specific function. RBE was calculated for SF value of 10%. To further explore the relative contributions of different DSB-based descriptors to the calculated survival response, the partial derivatives of $ - {\text{ ln}}S{F_{\mathrm{cal}}}$ with respect to each descriptor were computed analytically from the fitted polynomial model and evaluated at selected dose levels (1 Gy and 2 Gy).

### 2.4. Application in proton spread-out bragg peak (SOBP)

Using the resulting cell-specific function, we further explored the potential application of our approach in a proton SOBP irradiation case. Details of the simulation settings and SOBP configuration are provided in section S1.3. Estimated SF values were evaluated at various depths, and the RBE was calculated for SF value of 10%.

## 3. Results

### 3.1. DNA damage distributions for photon, proton, and carbon-ion cases

**Figure 2.**
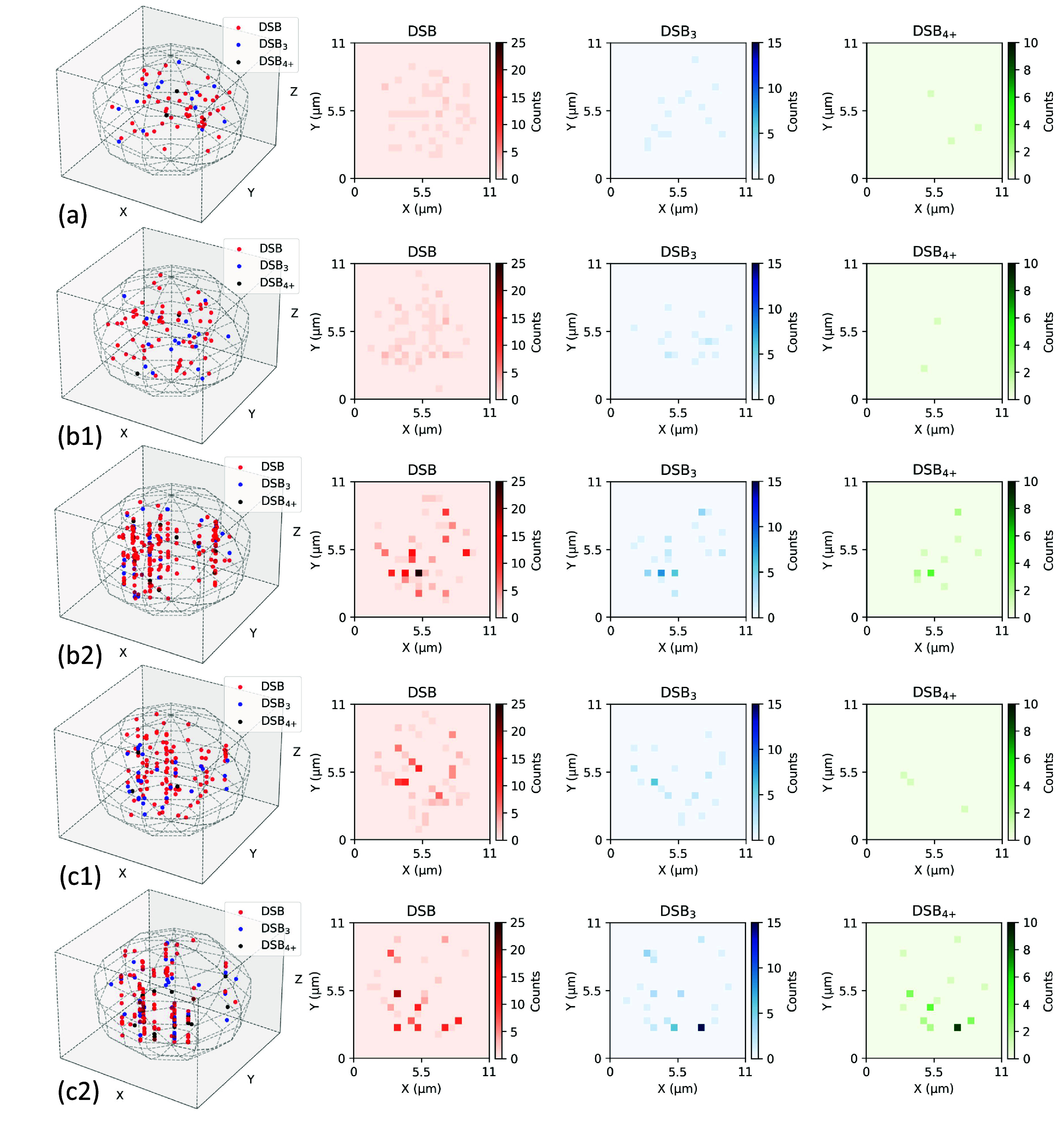
Spatial distributions of DSBs under 2 Gy dose in a cell nucleus for (a) ^137^Cs irradiation, proton irradiations of (b1) ${\bar y_d}$ = 2.0 keV *μ*m^−1^, (b2) 20.0 keV *μ*m^−1^, carbon–ion irradiations of (c1) ${\bar y_d}$ = 18.6 keV *μ*m^−1^ and (c2) 87.9 keV *μ*m^−1^. Four sub-figures in each row (from left to right) present the 3D spatial distribution of all DSBs and the 2D heatmaps of ${\mathrm{DSB}}$, ${\mathrm{DSB}}_3$, and ${\mathrm{DSB}_{4 + }}$ counts projected onto the *XY* plane.

Figure 2 shows the distribution of DSBs of various complexities under different irradiation conditions at a dose of 2 Gy. For proton irradiation at the relatively low ${\bar y_d}$ of 2.0 keV *μ*m^−1^ in figure 2(b1), the distribution of DSBs was similar to that of the photon case in figure 2(a). In both cases, DSBs were distributed relatively uniformly. As ${\bar y_d}{\text{ }}$ increases, for each type of particle, damage sites became more concentrated along the particle tracks, with a higher proportion of high-complexity damage occurring. Comparing figures 2(b2)–(c1), the spatial distribution of DSBs induced by 20.0 keV *μ*m^−1^ protons appeared denser than that induced by 18.6 keV *μ*m^−1^ carbon ions, despite their comparable ${\bar y_d}$.

**Figure 3.**
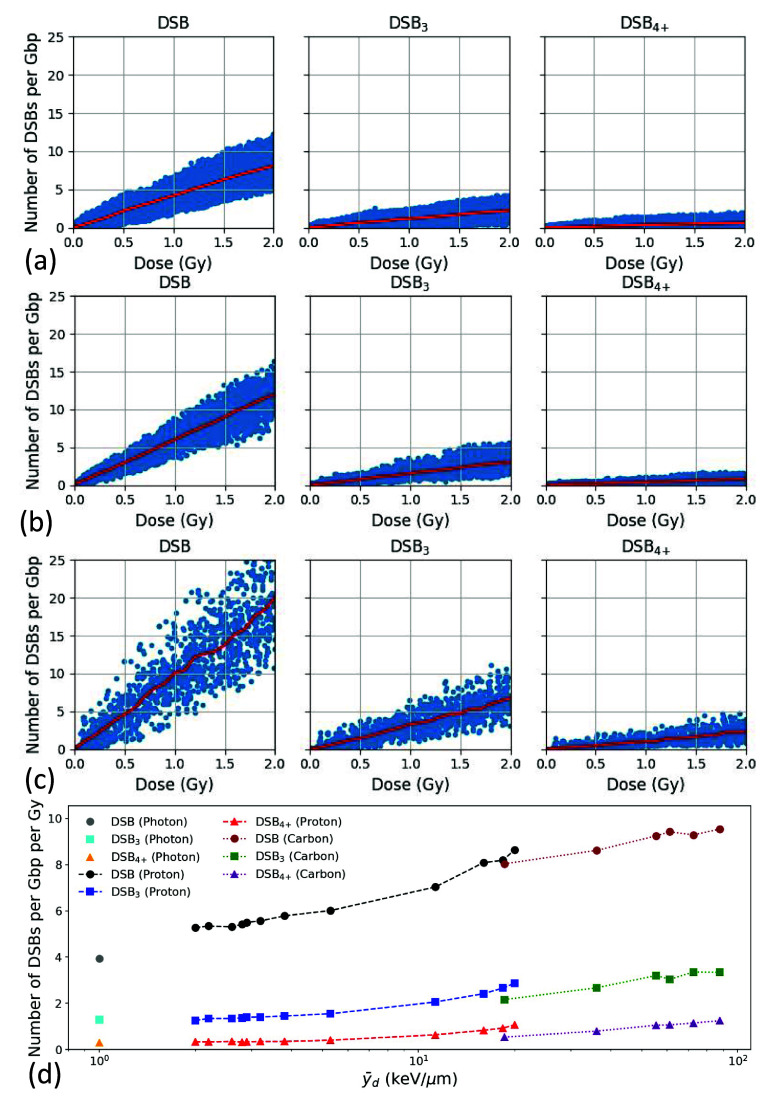
Numbers of DSBs as a function of dose for (a) ^137^Cs irradiation with ${\bar y_d}$ = 1.0 keV *μ*m^−1^, (b) proton irradiation with ${\bar y_d}$ = 5.3 keV *μ*m, and (c) carbon-ion irradiation with ${\bar y_d}$ = 72.6 keV *μ*m^−1^. Solid curves indicate averaged DSB numbers at each given dose level. (d) DSB yields of various complexities as functions of ${\bar y_d}$.

Figures 3(a)–(c) presents the number of DSBs of various complexities as a function of dose. At a given dose level, there was a spread of the number of DSBs among different simulations. We computed the average number of DSBs at each dose level. For ^137^Cs irradiation at 2 Gy, the average yields of ${\mathrm{DSB}}$, ${\mathrm{DSB}_3}$ and ${\mathrm{DSB}_{4 + }}$ (number per Gy per Gbp) were 4.03 ± 0.58, 1.15 ± 0.33 and 0.33 ± 0.14, respectively. For protons at ${\bar y_d}$ of 5.3 keV *μ*m^−1^, the corresponding yields were 5.99 ± 0.73, 1.49 ± 0.41, 0.42 ± 0.16, respectively. For carbon ions at ${\bar y_d}$ of 72.6 keV *μ*m^−1^, the yields reached 9.57 ± 2.45, 3.53 ± 1.08, 1.23 ± 0.40, respectively. We further plot DSB yields of different complexities against ${\bar y_d}$ in figure 3(d). Overall, the DSB yield increased with ${\bar y_d}$. Notably, at a given ${\bar y_d}$, the DSB yield also varied by particle type, producing the discontinuities observed at the junctions between particle types in the curves. This behavior arises directly from the distinct spatial patterns of energy deposition exhibited by different particles.

### 3.2. SF model fitting

**Figure 4.**
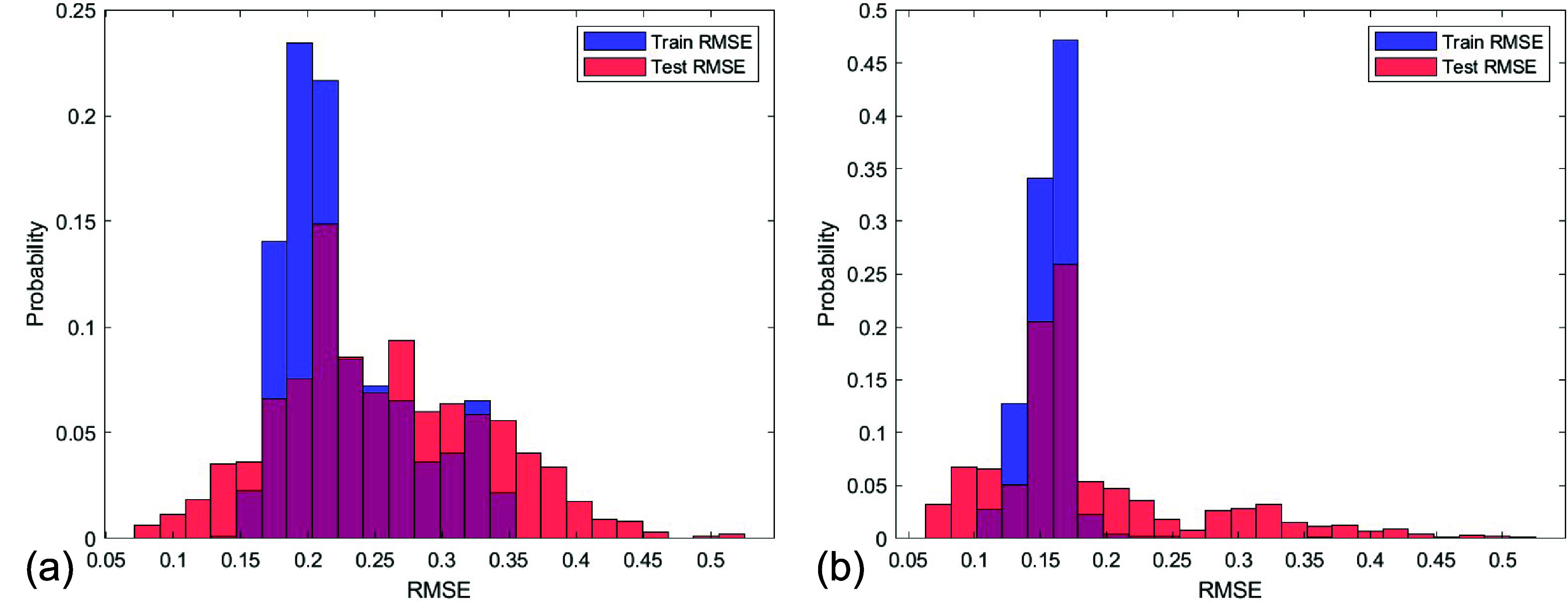
Evaluation of model validity for (a) H460 cells and (b) H1437 cells. RMSE histograms of model fitting (blue) and validation (red).

For the cross-validation study, figure 4 presents the RMSE distributions for H460 and H1437 cells. The distributions of the model fitting RMSE (blue bars) and the validation RMSE (red bars) approximately shared a similar shape, with average RMSEs of 0.227 ± 0.047 and 0.162 ± 0.020 for fitting, and 0.255 ± 0.077 and 0.178 ± 0.060 for validation, respectively. After verifying model validity, we used the model to fit all the experimental data, yielding the overall low RMSE values of 0.218 for H460 cells and 0.170 for H1437 cells.

**Figure 5.**
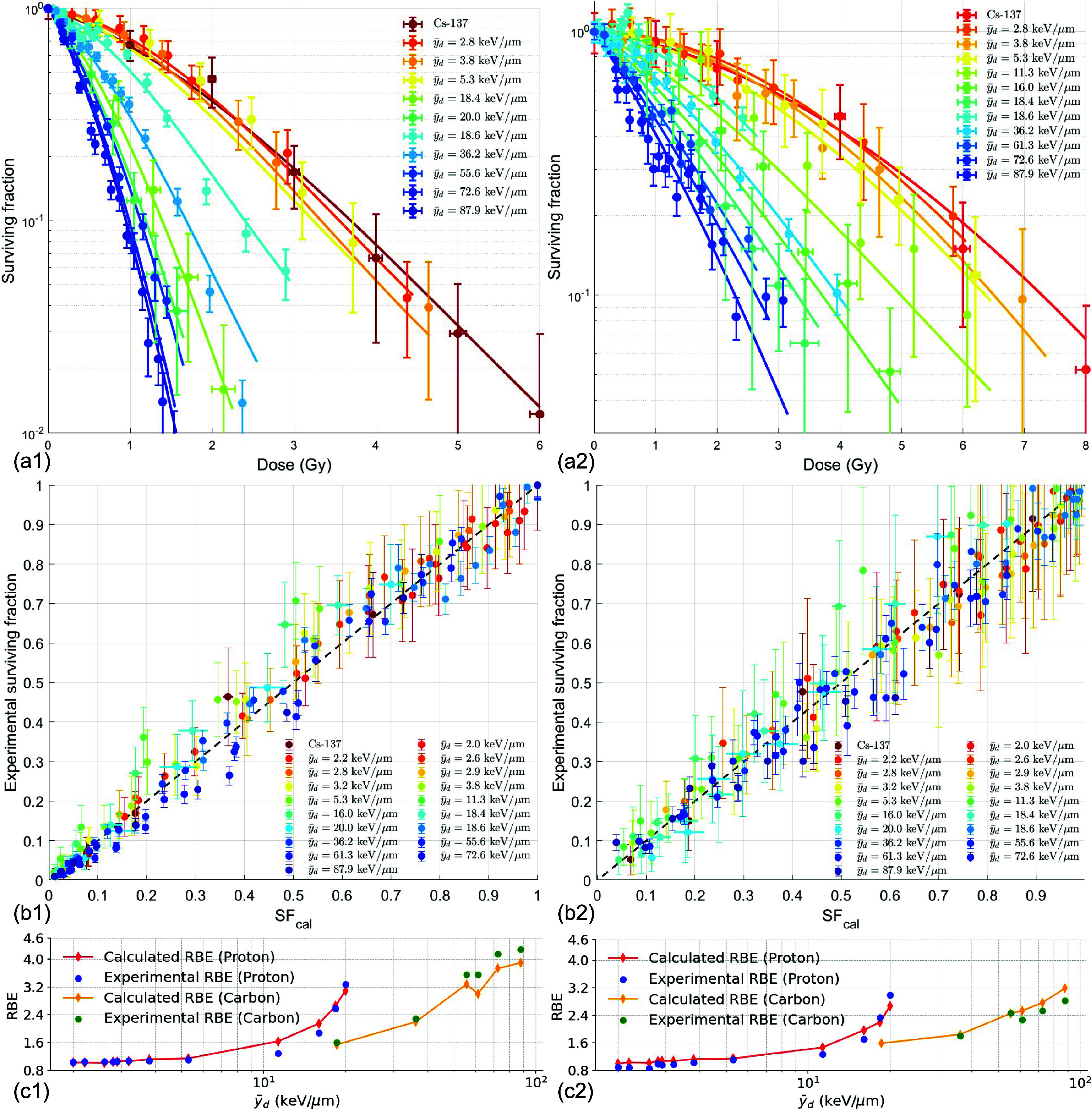
Model performance for H460 cells (a1)–(c1) and H1437 cells (a2)–(c2). (a1) and (a2) Comparison between model SF (solid lines) and experimental SF (symbols with errorbars). (b1) and (b2) Congregation of experimental points using $S{F_{cal}}.{\text{ }}$ (c1) and (c2) Calculated and experimental RBEs as functions of ${\bar y_d}$.

Fitting results for the two cell lines are shown in figure 5 and the polynomial coefficients are listed in table 1. When being plotted against dose, the fitted functions for different ${\bar y_d}$ are shown in figures 5(a1) and (a2), demonstrating good agreement with experimental data. We further plotted in figures 5(b1) and (b2) the experimental SF data against $S{F_{cal}}$. All data points, color-coded by ${\bar y_d}$, aligned closely with the diagonal dashed line, illustrating the consistency of $f\left( . \right)$ across irradiation conditions spanning different dose and ${\bar y_d}$ within the investigated dataset. The calculated RBE values as a function of ${\bar y_d}$ are shown in figures 5(c1) and (c2), where results were consistent with experimental values for both cell lines. The RBE curves exhibited discontinuities at the proton–carbon junction, consistent with experimental observations by Bronk *et al* (2020) and in the particle irradiation data ensemble database (Friedrich *et al* 2013). These discontinuities were caused by the distinct track structures of protons and carbon ions, which produce different DSB yields even at similar numerical values of ${\bar y_d}$, as shown in figure 3(d).

**Table 1.** Fitted coefficients ${a_1} - {a_{10}}$ of $f\left( . \right)$ for H460 and H1437 cells.

Cell line	${a_1}$	${a_2}$	${a_3}$	${a_4}$	${a_5}$	${a_6}$	${a_7}$	${a_8}$	${a_9}$	${a_{10}}$
H460	−0.142	0.351	1.312	0.058	0.428	1.308	−0.268	−0.360	0.451	−0.0001
H1437	−0.059	−0.060	1.240	0.008	0.267	1.361	−0.060	0.048	1.055	0

**Figure 6.**
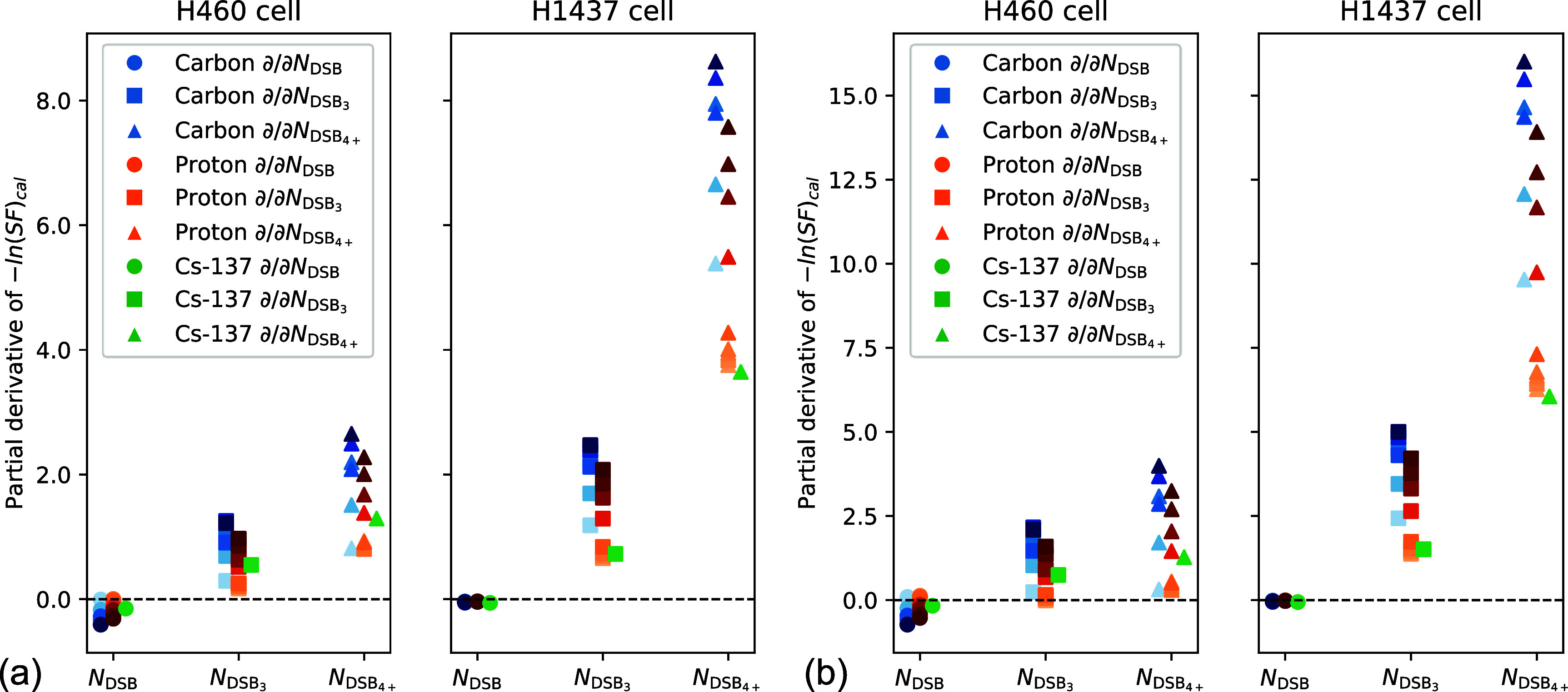
Partial derivatives of $ - {\text{ ln}}S{F_{cal}}$ with respect to the number of DNA damages of different complexities for H460 and H1437 cells at (a) 1 Gy and (b) 2 Gy. Within each particle type, irradiation conditions are ordered by increasing ${\bar y_d}$ (from lighter to darker colors).

Beyond fitting performance, we further analyzed the behavior of the fitted model through a derivative-based sensitivity analysis. Specifically, we evaluated the partial derivatives of $ - \ln S{F_{cal}}$ with respect to ${N_{\mathrm{DSB}}}$, ${N_{\mathrm{DSB}_3}}$, and ${N_{\mathrm{DSB}_{4 + }}}$. These quantities are analogous to the conventional definition of radiosensitivity with respect to dose, $ - \frac{{d\left( {{\mathrm{ln}}S{F_{cal}}} \right)}}{{dD}}$. Derivatives for all 19 experimental settings were evaluated and presented as individual data points in figure 6. The sensitivities associated with ${\mathrm{DSB}_3}$ and ${\mathrm{DSB}_{4 + }}$ were lower for H460 cells compared with H1437 cells, potentially reflecting differences in intrinsic radiosensitivity between the two cell lines. Among different irradiation types, carbon-ion irradiation exhibited a greater spread in derivative values, partly associated with the broader range of ${\bar y_d}$ and DSB yields. Within each particle type, irradiation conditions are ordered by increasing ${\bar y_d}$ (from lighter to darker colors), and the magnitude of the derivatives generally increased with ${\bar y_d}.$ This trend is qualitatively consistent with the reported behavior of the α parameter of the LQ model (Guan *et al* 2015, Bronk *et al* 2020). Similar trends were observed when the derivatives were evaluated at both 1 Gy and 2 Gy, indicating that the overall behavior was consistent across the selected dose levels.

### 3.3. RBE values in the proton SOBP case

**Figure 7.**
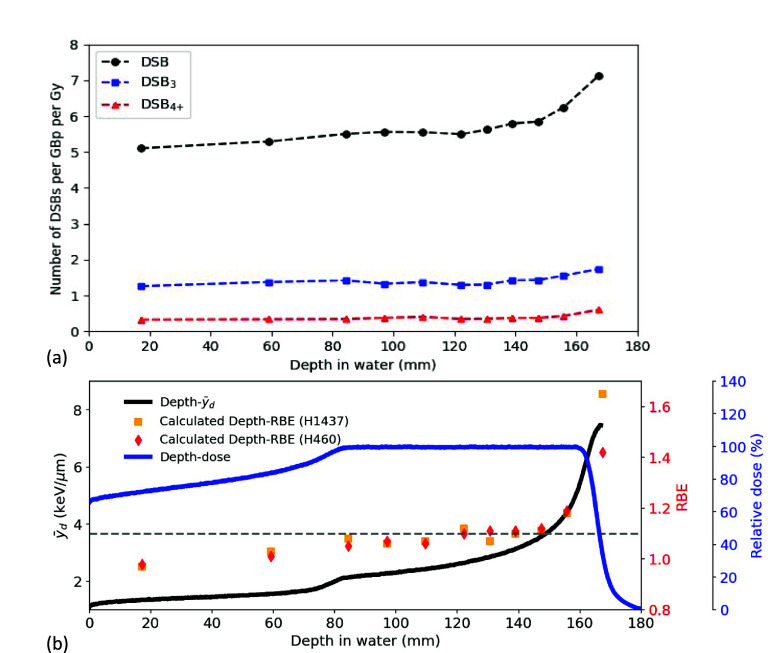
(a) DSB yields along the beam path of the proton SOBP case in a water phantom. (b) Depth- ${\bar y_d}$ curve (black), depth-dose curve (blue), and depth-RBE curves (red diamonds for H460 cells, orange squares for H1437 cells). The horizontal dashed line indicates the level of RBE 1.1.

The dose, ${\bar y_d}$, and DSB yields as functions of depth are presented in figure 7. While the dose remained nearly uniform within the SOBP, ${\bar y_d}$ increased slowly with depth both in the entrance region and inside the SOBP, and exhibited two jumps at both ends of the SOBP. For DSB yields, they remained relatively stable from the entrance to the center of SOBP (109.6 mm), with yields of ${\mathrm{DSB}}$, ${\mathrm{DSB}_3}$, and ${\mathrm{DSB}_{4 + }}$ changing from 5.51, 1.42, 0.34–5.56, 1.37, and 0.40 per Gy per Gbp. A significant increase was observed at the end (155.8 mm) and the distal fall-off region of SOBP (167.4 mm). The corresponding proton RBE calculated for H460 and H1437 cells are shown in figure 7(b). For the H460 cell, RBE values were 1.01 in the beam entrance region, 1.1 in the middle of the SOBP, further increased to 1.19 at its end, and 1.42 at the distal fall-off region. For the H1437 cell, RBE values were 1.03, 1.12, 1.18, and 1.65 at these respective depths.

## 4. Discussion

In this work, we computed DSBs of varying complexities and used them as independent variables to construct functions for describing cell SF within the investigated irradiation conditions. The functions were fitted using previously published SF data for two cell lines irradiated with photons, protons, and carbon ions. Cell-specific radiosensitivity is reflected by the corresponding function form. Validity of the model was demonstrated in the cross-validation study and by the feasibility of modeling SF across irradiations with various beam types, doses and LETs/lineal energies in a unified framework. Effectiveness of our model was further demonstrated in a representative proton SOBP case. The trend of increasing RBE values with depth depicted by our model aligned well with previous studies (Paganetti 2014).

Our approach to model the SF behavior decouples DNA damage formation from the subsequent DNA repair process. The DNA damage was computed by MMCS, whereas the impact of the repair process and the outcome of SF were described by the function. This idea was essential for the successful construction of $f\left( . \right)$ that provides a consistent description across particle type, dose, and ${\bar y_d}$ within the investigated conditions, as the impacts of these variables have been naturally coded in the model input variables, i.e. DSBs. It should be emphasized that although dose and ${\bar y_d}$ were presented in figure 5(a), these variables were not explicitly used in data fitting but rather implicitly via DSBs.

It is also worth noting that in figure 3(d), the yields of ${\mathrm{DSB}_3}$ and ${\mathrm{DSB}_{4 + }}$ for high-LET carbon ions appear comparable to those for lower-LET protons, which may seem counterintuitive. This observation reflects the limitation of LETs/lineal energies as a descriptor of radiation quality, as it does not uniquely characterize track structure and energy deposition topology across different particle types. A 20 keV *µ*m^−1^ proton corresponds to an average energy of approximately 4 MeV, placing it near the Bragg peak where low-energy secondary electrons deposit their energy very close to the primary track and produce highly localized nanometric energy density. A 100 keV *µ*m^−1^ carbon ion, by contrast, corresponds to an average energy of approximately 600 MeV (50 MeV u^−1^), a regime in which higher-energy delta electrons carry a substantial fraction of the deposited energy laterally away from the core track, dispersing energy from the nanometric volumes relevant to DNA clustering. Consequently, the local nanometric energy density that governs clustering behavior is not proportionally higher for the carbon ion despite its fivefold greater macroscopic LET, and a portion of the more spatially concentrated damage that does occur within the core may be grouped into single complex clusters rather than resolved as separate ${\mathrm{DSB}_3}$ and ${\mathrm{DSB}_{4 + }}$ events. As a result, ${\mathrm{DSB}_3}$ and ${\mathrm{DSB}_{4 + }}$ yields do not necessarily increase monotonically with LET across different particle types.

This interpretation is also influenced by the specific DSB complexity classification scheme adopted in the present work, which represents one possible modeling choice. Different clustered-damage classification schemes have been proposed in previous studies. For example, Sakata *et al* distinguished progressively complex DSB classes using a 10 bp DSB criterion together with additional separation rules (2020). Montgomery *et al* considered broader clustered lesions that include SSBs, base lesions, and DSB-containing clusters (2021). While alternative classification schemes may lead to differences in absolute yields of specific damage categories, the present framework primarily uses these descriptors to capture relative differences in damage patterns across irradiation conditions rather than absolute lesion counts. Therefore, the consistent application of the same classification scheme across all simulations ensures that the resulting survival model remains internally coherent. Sensitivity analyses using alternative classification schemes may be explored in future work.

The purpose of this study was not to exhaustively compare all possible functional families, but to establish and test a minimal DSB-based mapping framework. During model development we explored several alternative formulations, including linear and mixed linear-polynomial models, and ultimately employed a third-order polynomial representation for $f\left( . \right)$. The choice was partially motivated by the established ability of the LQ model to fit experimental data at each individual ${\bar y_d}$. Aiming at forming a unified model valid across all the ${\bar y_d}$ cases, the function $f\left( . \right)$ should have additional degrees of freedom beyond a purely quadratic model to capture the ${\bar y_d}$ dependence. As a first-order approximation, the $\beta $ coefficient in the LQ model for an individual ${\bar y_d}$ may be assumed to have a linear dependence on ${\bar y_d}$. Hence, a third-order polynomial provided a simple extension capable of capturing nonlinear dependencies while maintaining model interpretability. To maintain model simplicity and avoid overfitting, only a single third-order term $N_{\mathrm{DSB}}^3$ was included, as it accounts for the majority of DSBs.

Regularization plays a crucial role in our model fitting to ensure a smooth response of SF with respect to DSBs. This is particularly important given the uncertainty in experimental data, which could lead to overfit to noise, if only the data fidelity term were used in $L\left( \theta \right)$. Additionally, enforcing smoothness in SF model enhances model robustness, when dealing with DSBs computed via MC simulations, where uncertainty is inevitable.

MMCS is subject to systematic uncertainties, which can arise from various sources, including uncertainties in cross-section data, simulation parameters, and modeling of radiation physics and chemistry processes. One notable example is the challenge of accurately modeling low-energy secondary electrons, particularly those below approximately 10 eV (Chatzipapas *et al* 2020). However, due to the limited availability of experimental data on low-energy electron interactions with DNA, accurately modeling this process remains difficult. These inaccuracies can compromise the reliability of MMCS simulations, potentially leading to deviations in calculated DSB yields. Nonetheless, when constructing the SF model, since all simulations were performed under a consistent condition, we expect that the systematic uncertainty associated with MMCS may be inherently compensated for to a large extent during the data-fitting process, ensuring that the resulting SF modeling remains valid.

Despite the success, the present framework remains a simplified phenomenological representation of radiation response. DNA damage response is highly dynamic, with different repair pathways being differentially activated depending on damage complexity and cell status (Roobol *et al* 2020). Rather than simulating the temporal evolution of DNA repair and lesion processing, the present approach investigates whether initial DSB complexity alone contains sufficient predictive information for survival. Compared with more mechanistic multiscale frameworks such as MINAS TIRITH (Thibaut *et al* 2023) and Geant4-DNA-based approaches (Khanna *et al* 2025), which incorporate detailed representations of damage induction and downstream biological processing, the current model adopts a compact formulation that directly maps initial DNA damage complexity to survival outcomes without introducing additional biological processes beyond damage induction. These distinctions reflect a trade-off between mechanistic detail and model compactness. Mechanistic frameworks provide stronger biological interpretability by explicitly representing repair dynamics, stochastic population effects, and lesion evolution processes, whereas the present approach emphasizes a simplified and unified mapping between initial DNA damage complexity and cell survival across irradiation conditions. We therefore view these approaches as complementary rather than competing. Future mechanistic models incorporating explicit repair dynamics may still benefit from MMCS-derived DSB descriptors as physically grounded representations of irradiation-induced damage complexity.

Within this phenomenological framework, the sensitivity trends shown in figure 6 provide additional insight into the behavior of the fitted model. The comparison among cell lines aligns with the radiosensitivity-specific proteomic network studies (Zhu *et al* 2022), which indicate that H460 is more resistant to DNA damages of more complexities. Nevertheless, these derivatives are inferred from a phenomenological polynomial model rather than derived from mechanistic repair kinetics and thus should not be over-interpreted biologically. A more rigorous investigation incorporating additional cell lines and mechanistic repair modeling would be necessary to establish a robust biological interpretation of the observed differences.

For future work, the assumption of a cell-specific SF model driven by initial DNA damage warrants further examination. Model performance should be evaluated under additional irradiation conditions, including other particle types and broader LET ranges, to assess generalizability. Currently, analysis was restricted to datasets from the same experimental group to minimize inter-laboratory variability in radiosensitivity, and helium as well as high lineal energy carbon-ion conditions were excluded because of substantial experimental uncertainty. At very high lineal energy, experimental SF data exhibits increased non-monotonic behavior with dose and even wrong values exceeding unity (Bronk *et al* 2020). To avoid introducing bias from such effects, model fitting was restricted to irradiation conditions with stable and well-behaved survival trends. Consequently, the applicability of the present model to the overkill regime remains uncertain in the absence of sufficient and stable fitting data with high LET. Future studies incorporating more high-quality data across multiple cell lines and experimental platforms will be essential to determine whether the current model can accurately describe survival behaviors across a broader range of particle types and LET conditions. In addition, the relationship between DNA-damage metrics and detailed microdosimetric spectra should be investigated directly, rather than relying on averaged quantities such as the dose-mean lineal energy, ${\bar y_d}$ (Guan *et al* 2024).

Beyond further biological validation, another important direction is to address the challenges to facilitate the translation of this modeling approach to clinical treatment planning. At present, the proposed framework should be regarded primarily as a methodological and radiobiological modeling approach that demonstrates potential relevance to particle therapy, rather than implying readiness for direct clinical treatment-planning application. A major challenge for clinical translation is the computational cost of MMCS simulations. Voxel-level SF estimation in treatment planning would require prohibitively large numbers of DNA-damage calculations. A clinically viable strategy must therefore avoid real-time MMCS simulations. One potential solution is to develop a mapping between particle fluence and DSB yields, enabling efficient voxel-wise computation. Ultimately, successful translation to clinical workflows will require both extensive validation across diverse radiation qualities and biological systems and the development of computationally tractable surrogate formulations. These advances will determine whether DNA-damage–driven SF modeling can provide a robust biologically informed alternative to conventional dose-based metrics.

## 5. Conclusion

In conclusion, we developed a cell-specific SF model framework using initial DSBs of varying complexities as input variables. By linking MMCS–computed DNA damage to cellular survival, the proposed framework provides a unified description of photon, proton, and carbon-ion irradiations within the investigated irradiation conditions. This approach grounds SF modeling in microscopic DNA damage metrics that inherently reflect radiation track structure, and provides a physics-informed framework for relating DNA damage to cell survival, with potential future applications in particle therapy.

## Data Availability

All data that support the findings of this study are included within the article (and any supplementary information files). Supplementary material available at https://doi.org/10.1088/1361-6560/ae7baa/data1.
